# Immunomodulation and Regenerative Capacity of MSCs for Long-COVID

**DOI:** 10.3390/ijms222212421

**Published:** 2021-11-17

**Authors:** Xin Ya Loke, Siti A. M. Imran, Gee Jun Tye, Wan Safwani Wan Kamarul Zaman, Fazlina Nordin

**Affiliations:** 1 Centre for Tissue Engineering and Regenerative Medicine (CTERM), Faculty of Medicine, Universiti Kebangsaan Malaysia, Jalan Yaacob Latiff, Bandar Tun Razak, Cheras, Kuala Lumpur 56000, Malaysia; xinya7733@outlook.com (X.Y.L.); siti.imran@ukm.edu.my (S.A.M.I.); 2 Institute for Research in Molecular Medicine (INFORMM), Universiti Sains Malaysia, Gelugor 11800, Malaysia; geejun@usm.my; 3 Department of Biomedical Engineering, Faculty of Engineering, Universiti Malaya, Kuala Lumpur 50603, Malaysia; wansafwani@um.edu.my; 4 Centre for Innovation in Medical Engineering (CIME), Department of Biomedical Engineering, Faculty of Engineering, Universiti Malaya, Kuala Lumpur 50603, Malaysia

**Keywords:** MSCs, long-COVID, immunomodulation, regeneration

## Abstract

The rapid mutation of the SARS-CoV-2 virus is now a major concern with no effective drugs and treatments. The severity of the disease is linked to the induction of a cytokine storm that promotes extensive inflammation in the lung, leading to many acute lung injuries, pulmonary edema, and eventually death. Mesenchymal stem cells (MSCs) might prove to be a treatment option as they have immunomodulation and regenerative properties. Clinical trials utilizing MSCs in treating acute lung injury (ALI) or acute respiratory distress syndrome (ARDS) have provided a basis in treating post-COVID-19 patients. In this review, we discussed the effects of MSCs as an immunomodulator to reduce the severity and death in patients with COVID-19, including the usage of MSCs as an alternative regenerative therapy in post-COVID-19 patients. This review also includes the current clinical trials in utilizing MSCs and their potential future utilization for long-COVID treatments.

## 1. Introduction

Coronavirus disease 2019 (COVID-19) is a newly discovered, rampantly spreading disease. According to the World Health Organization (WHO) [1], a total of 218,205,951 cases of COVID-19 were confirmed and 4,526,583 deaths were reported as of 2 September 2021 globally. In December 2019, this newly emerged disease was first identified in Wuhan City, Hubei Province of China, from a seafood wholesale wet market [2,3]. A mysterious pneumonia case characterized by fever, cough, and fatigue was identified, and later its causing agents were identified as a novel β-coronavirus, named severe acute respiratory syndrome coronavirus 2 (SARS-CoV-2). Since the outbreak of the disease, WHO had declared it as a health emergency in January of 2020, and as a global pandemic in March 2020 [2,4,5]. This is the first pandemic caused by coronavirus (CoV) [6].

In this situation, prevention steps should be taken by every individual to protect oneself and others. The Centers for Diseases Control and Prevention (CDC) [7] suggested that wearing a mask in public places, washing hands regularly with soap, and practicing social distancing are among the steps to lower down the disease’s transmission rate. If an individual is infected, quarantine is required to prevent the further spreading of the virus, and if not contained, can eventually lead to the formation of new clusters. For patients with severe infection, hospitalization is required for intensive care, with some requiring ventilators to maintain the saturation of oxygen in the body [8].

As viruses are prone to mutation, as of 31 August 2021, 12 variants of SARS-CoV-2 were identified and classified into two categories: variant of interest (VOI) and variant of concern (VOC). The mortality of each variant appears to be independent of the virus strains. A VOC is high in transmissibility and fatality, causing a decrease in vaccine effectiveness. These variants will increase their virulence and change their clinical diseases presentation, causing false results with a clinical test. As these variants can decrease the effectiveness of vaccines developed, the public health and social measures, therapeutics, and diagnosis are also made less effective in preventing infection by these variants of the virus. VOI contains the capability to escape diagnosis and the immune system due to the changes in their genetic composition, affecting the disease’s capability and transmissibility [9]. These mutations were also attributed to solid community transmission, resulting in COVID-19 clusters with rising prevalence and case numbers over time. These mutations were thought to be an emerging threat to worldwide public health. The variants were labeled by WHO (Table 1) in collaboration with many researchers, experts, and national authorities to globally monitor and respond to the changes of the COVID-19 pandemic [10].

### 1.1. Mechanism of COVID-19

**Figure 1:**
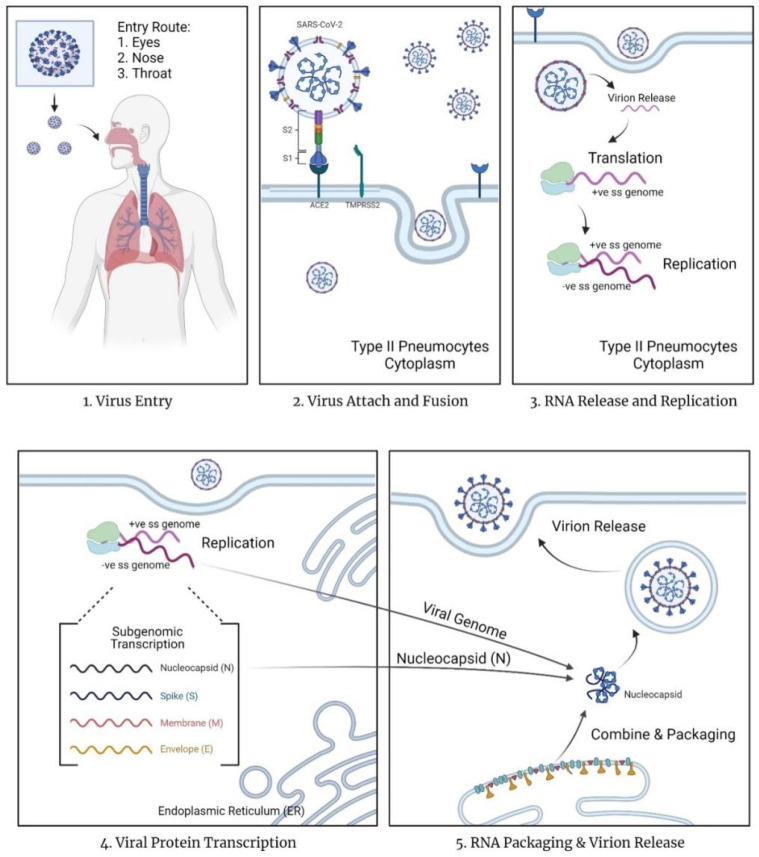
The mechanism of entry and replication of coronavirus diseases 2019 in host cells. Created with BioRender.com.

The mechanism of action for COVID-19 currently remains unknown [11]. In contrast, the mechanism of entry and replication is known (Figure 1). Since COVID-19 is an airborne infectious disease, it enters via the eyes or nasopharyngeal tract and replicates within the body by turning the host cells into virus factories. According to Çetin & Topçul [6], SARS-CoV-2 may pass through the mucous membrane and enter the lungs via the respiratory tract. SARS-CoV-2 contains homotrimer spike proteins (S proteins), which are class I fusion glycoprotein with two distinct parts, S1 and S2 subunits [12]. The spike proteins will attach to the surface cell receptors, angiotensin-converting enzyme 2 (ACE 2) present on type 2 pneumocytes in the alveoli of the lungs [11,12,13]. This binding reaction allows the virus to infect lung cells. The spike protein of the virus will be subjected to a two-step proteolytic cleavage process by the lung cells between the S1 and S2 subunits [11,12]. The two steps involved in the cleavage at the boundary of the S1 and S2 subunit, and the conserved site of upstream fusion peptide (S2) [11,14]. The S2 domain will release a fusion peptide after cleavage. This will trigger the event of the membrane fusion mechanism, further promoting the endocytosis of the virus entering the cytoplasm of epithelial cells.

Based on Simmons et al. [15] the virus employs unique steps in membrane fusion: receptor-binding, inducing conformational changes of glycoproteins of spikes (S), cathepsin L proteolysis via intracellular proteases, and further activation within endosome for the membrane fusion mechanism. The endosomes will open and release the virus, allowing the uncoating of nucleocapsid (N) protein by proteasomes. Once the virus contents are present in the cytoplasm of the cells, the replication and transcription process start via the replication/transcription complex (RTC). The viral genetic material released in the cytoplasm is a positive single-stranded RNA (ssRNA). This positive sense of ssRNA is translated, generating replicase proteins. The replicase proteins will then generate full-length negative-sense RNAs, with the positive single-stranded RNA as the template.

Within the cytoplasm, structural viral proteins such as proteins M, S, and E are also translated and synthesized. The proteins will be inserted into the endoplasmic reticulum (ER), and then transferred to the endoplasmic reticulum-Golgi intermediate compartment (ERGIC) [11]. N protein stimulates the encapsidation of the replicate genome, forming the nucleocapsid of the virus. This nucleocapsid will bind with the ERGIC for self-assembly into the new virions. Eventually, the new virions are transported to the cell wall via smooth-walled vesicles and released via exocytosis. The released virus will continue infecting other cells, turning them into a new replicating factory. Simultaneously, the stress of viral production leads to cell death.

### 1.2. Severity of the COVID-19

COVID-19 severity depends on several factors. The symptoms are clinically divided into five categories, some are asymptomatic or paucisymptomatic individuals, some with mild symptoms, moderate symptoms, severe symptoms, and critical symptoms [16] According to Moll et al. [17] most COVID-19 patients are asymptomatic to flu-like symptoms, approximately 17% of the cases are with mild to severe symptoms that lead to fatal outcomes. Hence, scientists suggested a prevailing theory stating that the severity of the diseases might be due to the effectiveness of the immune system of an individual in fighting against the diseases and releasing the antibodies. The disease tolerance of an individual that can accommodate and make internal adjustments makes the infection severity different for different individuals.

Mutations of the coronavirus strains affect the severity of diseases. According to Reardon [18], the virus was in a stable state when it first emerged and not readily mutated. However, this virus acquired a random mutation in a minor state when it jumped from animals to humans, becoming a zoonotic disease [19]. The mutations of the virus drive major changes in its alterations and contagiousness. These mutations allow the transmission of viruses that evade the immune system of the host efficiently. This will lead to the increased severity of the COVID-19 infection as the antibodies of individuals cannot completely neutralize the virus, resulting in the immune escape ability of the coronavirus variants. The severity of the disease increases when the mutation of the virus continues, and possibilities exist for the virus to evolve into a vaccine-resistant phenotype [20].

Infection dose also changes the severity of the virus. It was long known that the dose of exposure to infectious diseases is important in determining the disease’s severity. As the dose of exposure to the infectious agent is low, an individual might exhibit no or mild symptoms. However, highly infectious individuals will exhibit severe symptoms and increase the severity of diseases. With a highly infectious dose, patients will develop severe and critical symptoms, and associated complications as an antiviral-based strategy does not work for them [21]. Hospitalization and intensive care are required for these patients to prevent loss of life.

Individuals with at least one major underlying medical condition also affect the severity of coronavirus diseases [22]. These conditions weaken infection resistance, allowing the virus to infect easily. Medical conditions such as asthma and chronic obstructive pulmonary disorder cause the reduction of lung function which will further increase lung inflammation susceptibility. Pelzman [22] also stated that drugs used in treating high blood pressure may boost the level of angiotensin-converting enzyme 2 (ACE2), increasing the entry rate of the virus. Individuals with such conditions will commonly exhibit mild or severe symptoms as they are less resistant to diseases.

Age is also a risk factor that directly correlates to the severity of COVID-19. Individuals aged above their 50s are more likely to suffer from severe symptoms, be hospitalized, and adversely die [23]. According to an analysis of Chinese data and a study of early U.S. cases by the CDC [22], both study and analysis had similar findings, stating that the chances of death in confirmed COVID-19 cases estimated for patients 80 and older are more than 13%. In comparison to patients in their 30s, there are only 0.15% of deaths and virtually zero for patients under 20.

### 1.3. Current Problems with COVID-19 Treatment and Vaccines

To date, there is no effective cure for COVID-19. Recovery solely depends on the immunity of the individuals. Vaccines are developed as a prevention strategy to cope with the pandemic [24]. Vaccines allow the immune system to recognize the virus before infection and generate a memory immune response that would prevent infection or severe disease. According to Saha et al. [25], there were a total of 146 COVID-19 vaccines including live-attenuated vaccine, inactivated, or killed vaccine, subunit vaccine, and nucleic acid-based vaccine currently engaged in clinical trials. Moreover, it is believed that the number of vaccine candidates will increase in the future due to the increase in VOC/VOI. The first vaccine for COVID-19, which is now approved by the FDA, is the Pfizer-BioNTech COVID-19 Vaccine, an mRNA vaccine authorized for individuals above 12 years of age [26]. There are also other emergency use approved vaccines such as Sinovac-CoronaVac (inactivated vaccine), Novavax (subunit vaccine), and Oxford Astra-Zeneca (viral vector vaccine) which have proven to be effective in preventing severe COVID-19.

According to WHO [27], all vaccines that had been approved for emergency use listed have their quality, safety, and efficacy tested. Every vaccine that is approved has an efficacy of 50% and above. The ongoing safety and effectiveness of the vaccines were also monitored from time to time. Vaccines provide strong protection but require time to be fully functional. WHO also initiated a COVAX vaccine portfolio that involves both the government and the manufacturers to produce COVID-19 vaccines [13]. This global program will ensure the COVID-19 vaccines are fairly distributed among the higher and lower-income countries as prevention and preparation for recovery from the current pandemic. However, there are still breakthrough infections where vaccinated individuals might be infected albeit lower severity. In this regard, supplementary therapeutic interventions may be required.

### 1.4. Cytokine Storm and Immunomodulatory Mechanism

COVID-19 infection can give rise to cytokine storms in the lungs of moderate and severe patients. A cytokine storm, also known as hypercytokinemia, is an uncontrollable inflammatory response by the immune system [28]. This aggressive immune response triggers the release of a large amount of proinflammatory and anti-inflammatory cytokines, a diverse group of proteins secreted by the cells for intercellular communication and signaling, and chemokines (Table 2), disrupting the balance of the inflammatory response, physiological and pathophysiological [6,29,30]. The positive feedback mechanism that occurs in immune cells alternates this beneficial system into destructive ones. Zhao [31] stated that a cytokine storm is associated with the severity of COVID-19. Multiple immune-active molecules will exert their combination effects resulting in acute respiratory distress syndrome (ARDS) when there is the continuance of the cytokine storm in a hyperactive state. This also correlates directly with multiple organ failures such as edema, dysfunction of air exchange, acute cardiac injury, and secondary infection, leading to death in severe COVID-19 infection, prompting an unfavorable prognosis [32,33].

Cytokine storm should not be neglected in COVID-19 infection. Therefore, an immunomodulation approach by the stem cell is highly advocated as a main therapeutic strategy to downregulate the effect of a cytokine storm [31,47]. Immunomodulation mechanisms can alter the biological and immune response of the organisms. With the ability to alter the immune response of the host cells, regulation of the inflammatory responses within the host is required to prevent exaggerated cell damage caused by cytokine storms. Stem cells exhibit two types of immunomodulatory properties: immunosuppression and immunostimulation. Immunosuppression properties of stem cells contribute largely to the immunomodulation of host cells.

In COVID-19, the targeted organ is the respiratory organ, the lungs. Usage of stem cells such as mesenchymal stem cells (MSCs) for immunomodulation therapy is beneficial to regulate the proliferation, activation, and effector functions of all immune cells [6]. Stem cells are capable of immune-suppressing the activity and cytokine secretion of neutrophils and macrophages [48]. MSCs can also modulate innate and adaptive immune cells by enhancing anti-inflammatory pathways in the injured organ. It will also decrease proinflammatory mediators (IL-1β, TNF-α, IFN-γ, IL-6) and increase anti-inflammatory cytokines (IL-10, basic fibroblast growth factor (bFGF), TGF-α, TGF-β) in preventing cell damage [49].

MSCs contain specific mechanisms of action that are possible for prophylaxis or treatment following infection to regenerate damaged organs. First, MSCs secretes keratinocyte growth factors (KGF). This factor helps in clearing the alveolar fluid induced by excessive inflammation in the injured alveolar. It will upregulate key epithelial sodium channel gene expression and Na-K-ATPase activity or increase the trafficking of epithelial sodium channel proteins to the apical membrane. MSCs also secrete angiopoietin-1 (Ang1) that reduces lung and endothelial permeability through enhanced endothelial survival and vascular stabilization, preservation of cell adhesion molecules and cell junctions, and the prevention of actin “stress fibre” formation. These mechanisms and regulations allow MSCs to exhibit their regenerative properties in repairing damaged organs.

With the unraveling of COVID-19, cytokine storm, and the immunomodulatory function of MSCs, MSCs are currently being recognized as potential cell-based therapeutic targets for COVID-19. Hence, in this review, the possible mechanisms of MSCs’ immunomodulatory, regenerative mechanisms, and current clinical trials and their challenges will be deduced for potential COVID-19 treatments.

## 2. Immunomodulatory Effects of MSCs in COVID-19

**Figure 2:**
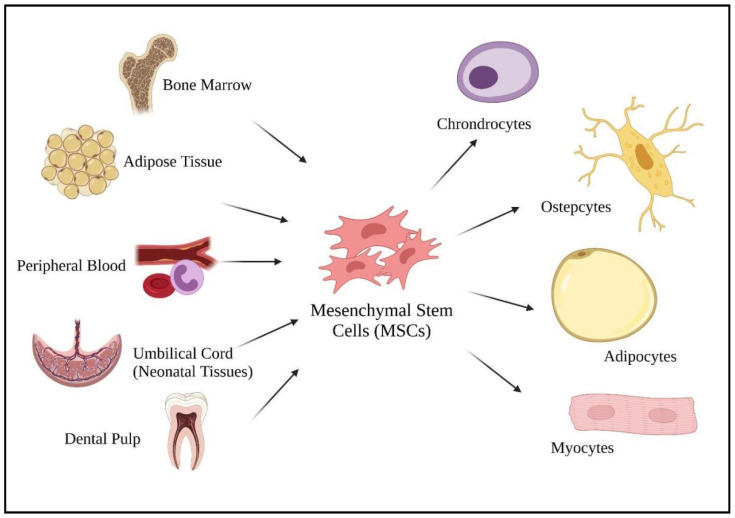
Sources of mesenchymal stem cells and their differentiated lineage. Created with BioRender.com.

Mesenchymal stem cells (MSCs) are well known for their promising cell-based therapies in infectious diseases due to their immunomodulatory potentials in managing inflammatory diseases [6,50]. According to Weiss and Dahlke [51], MSCs can be harvested from various adult tissues, from bone marrow to adipose tissue, dental pulp, peripheral blood, as well as from neonatal tissues (umbilical cord) (Figure 2). Compared to fetal and umbilical stem cells, adult stem cells are preferable as there are fewer ethical issues, widespread availability, and are high in clinical applications. As a multipotent cell, MSCs give rise to other descendent lineages such as chondrocytes, osteocytes, adipocytes, and myocytes (Figure 2). The ability to differentiate into various descendant cell lineages allows the use of stem cell therapies in treating chronic diseases, such as COVID-19.

### 2.1. Immunological Mechanism during COVID-19

As mentioned previously, the best defense against COVID-19 is the immune system, as it sets up the natural ability of the body to defend against pathogens. COVID-19 will not affect us as long as our body has good immunity [52]. However, when an individual is infected by COVID-19, the virus will induce cytokine secretion and localize inflammation [53]. The over-response of the immune system leads to the release of cytokines, chemokines, and other immune effector cells that are proinflammatory, causing cell damage. In this situation, MSCs will exhibit their immunosuppression properties to suppress the overreaction of the immune system towards COVID-19. Kavianpour et al. [53] also stated that the MSCs will locate at the inflamed lung tissues and secrete factors that can modulate the immune system. This will enable the prevention of oxidative stress (ROS) and fibrosis of the lung tissue.

In dealing with COVID-19, both innate and adaptive immunity are involved. According to WHO [54], the immunological mechanism of COVID-19 infection involves two phases. The first phase is the activation of innate immunity. Similar to other infectious diseases, the first and second lines of defense will restrict the entry of the SARS-CoV-2 virus into the host cells. The immune response will secrete interferon and chemical substances such as cytokines. Interferon will interfere with the replication of viruses in the cells while cytokines cause inflammatory reactions. Specifically, the innate immune response towards the infection mainly depends on the interferon type I protein with its downstream signaling. The viral RNA of the virus will act as the pathogen-associated molecular molecules (PAMPs) recognize by the toll-like RNA receptors (TLR-3 and TLR-7) and cytosolic RNA sensor (RIG-I/MDA5) [55]. NF-κB and interferon regulatory transcription factor-3 (IRF-3) will be activated and translocated into the nucleus. Inside the nucleus, the expression of IFN type I and proinflammatory cytokines is induced, constructed as the first-line defense against virus entry.

Generally, the infection stops at the first line of defense if the individual immunity is strong enough, or if the viral load is low. This IFN type I response is vital for viral suppression. However, this response is not effective in dealing with SARS-CoV-2 as the response is suppressed, and the virus will induce their programmed cell death, apoptosis. Without it, the virus continues replicating and causes delayed IFN type I response, heralding the influx of macrophages and neutrophils as a source of inflammation and Th1 immune response [55].

When the virus encounters a weaker immune system, clearance does not occur, resulting in the stimulation of adaptive immunity. Adaptive immunity via T cells and B cells would then provide the protection required. T cells are regulated by the antigen-presenting cells (APCs) by engendering the cytokine environment. It recognizes cells infected with the virus and rapidly differentiates and proliferates. CD8+ cytotoxic T cells clear virus-infected cells leading to reduced viral load. CD4+ helper T cells produce proinflammatory cytokines and mediators to further strengthen the response, including stimulating B-cells. B-cell produces antibodies against the virus leading to high amounts of neutralizing antibodies and Th2 cytokines (IL-4, IL-5 and IL-10).

### 2.2. Immunomodulation Mechanism of MSCs Involving Molecular Signaling

The immunomodulation ability of the MSCs can be exerted in various ways: soluble factors (paracrine interaction), cell–cell contact, and extracellular vesicles (EVs) [56,57]. The effect of the mechanism is exhibited either individually or in combination with various biomolecules by MSCs on the immune cells such as macrophages and neutrophils, DCs, T cells, B cells, and NK cells (Table 3) [6,58].

#### 2.2.1. Immunomodulating Effects of MSCs on Macrophages and Neutrophils

Based on a study by Mallis et al. [59], MSCs can modulate the macrophage phenotype (M1/M2) via cell–cell or paracrine interaction. Macrophages and neutrophils are important in the antigen presentation process to dendritic cells (DCs) that will stimulate cellular immunity. M1 phenotype macrophages, which are classically activated, are responsible for phagocytosis of pathogens, and present their antigen epitopes to DCs. This process will stimulate the release of cytokines (TNF-α, ΙL-1β, ΙL-1α, IL-6, IL-12) that cause inflammation by M1 macrophages and activate the Th1 immune response. Alternately, M2 macrophages (alternatively activated) promote the Th2 immune response. Th2 immune response counteracts with Th1 immune response, having immunosuppressive properties. These cells are high in anti-inflammatory molecules, associated with tissue repairs and cell apoptosis clearance. This cell–cell interaction enables MSCs modulation of macrophage phenotypes.

The presence of IFN-γ activates MSCs and results in the production of TNF-α, MCP1, and IL-1β. These secreted soluble factors can advance the phenotype of M1 macrophages. On the other hand, MSCs also express prostaglandins E2 (PGE2), which will induce an M2 macrophage phenotype switch. A study has shown that the activation of signal transducer transcription-3 (STAT3) occurs via cell–cell interactions between MCSs and the macrophages [60]. STAT3 transcription factors are responsible for IL-10 production that promote immunosuppressive functions.

#### 2.2.2. Immunomodulating Effects of MSCs on DCs

MSCs can interfere with the maturation of DCs via the production of soluble factors. Factors such as TNF-α, IL-1β, and IL-6 produced by M1 macrophages and IFN-γ will activate the MSCs and drive the maturation of DCs. Alternatively, PGE2 secreted by the activated MSCs is vital in the inhibition of DCs maturation. This soluble factor is crucial in preventing further damage to the lung cells and tissues. According to a study by Liu et al. and Sadeghi et al. [57,61], inhibition of DCs maturation by PGE2 interfere with T cells responses, as there are lower levels of CD38, CD80, CD86, IL-12, and IL-6 which are important for T cells activation and lead to cytokine storm. PGE 2 also lowers the migratory ability of DCs via CCR7–CCL21 interaction. Moreover, Liu et al. [61] also suggested that a stimulating gene produced by TNF-α produced by MSCs can also suppress DCs maturation by inactivating the signaling cascades mediated by mitogen-activated protein kinase (MAPK) and nuclear factor-kappa B (NF-κB). HLA-G soluble factors also prevent the differentiation of monocytes to DCs by blocking the secretion of cytokines such as TNF-α, ΙL1-α, β, IL-6, IL-7, IL-8, IL-9, GM-CSF, and IFN-γ. Furthermore, extracellular vesicles (EVs) that contain specific miRNAs such as miR-21-5p, miR-142-3p, miR-223-3p, and miR-126-3p also contribute to the inhibitory of maturation of DCs.

#### 2.2.3. Immunomodulating Effects of MSCs on T Cells and B Cells

There are several ways of MSCs immunomodulation via regulation of T cells. For example, secretion of molecules that affect the T cells responses and the cell–cell contact positively and negatively to inhibit the T cells proliferation. The production of PGE2, indoleamine-2,3-dioxygenase (IDO), TGF-β, and hepatocyte growth factor (HGF) will effectively inhibit the proliferation of T cells [62]. According to Mallis et al. [59], PGE2 is a prostanoid, responsible for T cells activation by the production of cAMP and can exert immunosuppressive properties on T cells. The cAMP produced can downregulate the IL-2 and IL-2R expression which will contribute to the activation of T cells receptors. PGE2 will also inactivate the T cells by negatively regulating the phosphatidylinositol hydrolysis and the diacylglycerol and inositol phosphate (IP) production. The immunosuppression mechanism of MSCs via T cells also can be shown by the T cells polarizing that will promote the Th2 immune response and orchestrate regulatory T (Treg) responses. Moreover, the immunosuppressive response also happens when blockage of metabolism by IDO from tryptophan to kynurenine is essential for T cell cycles [62]. When IDO is combined with TGF-β1 and HGF, T cells proliferation will be suppressed. The NO signaling pathway also suppressed the T cells response by activating the transcription 5 phosphorylation, resulting in the inhibition of TCR-mediated T cells proliferation and inflammatory cytokine production [59,62]. Galectins 1 and 3 also effectively suppress the proliferation of T cells proliferation by preventing the clustering of TCR via a crosslink interaction mechanism. There are also soluble HLA-G factors that can suppress the proliferation of hyperactive T cells with the presence of IDO and IL-10.

For cell–cell interactions, MSCs regulate their immunomodulatory properties by T cell apoptosis. According to Mallis et al. [59], Fas/Fas ligand death signaling pathway can lead to cell death of T cells. This action happens by the downstream activation of the Fas-associated death domain and caspases. MSCs will express Fas ligands when encountering inflammation stimuli. The Fas ligand binds to the Fas receptors of the hyperactivated T cells. TNF-related apoptosis-inducing ligand (TRAIL)/death receptors (DRs) signaling pathway is another potential activator for the Fas-associated death domain. IFN-γ causes the high production of TRAIL and binds to DRs on T cells, causing apoptosis. Programmed death ligand-1 (PD-L1)/programmed death-1 (PD-1) interaction also reduces T cells proliferation. The inhibition of MAPK due to the binding of PD-L1 to the PD-1 molecules of T cells, followed by Src-homology 2 domain containing protein tyrosine phosphatases (SHP)-1 and SHP-2 phosphorylation inhibits cellular proliferation.

MSCs immunomodulate B cells via secretion of soluble factors and cell-cell interaction. Both of these pathways are similar to the T cell’s regulation response. IDO, PGE2, the production of TGF-β1, and HGF can lead to cell cycle arrest of B cells. Fas/Fas ligand, TRAIL/DR death signaling, and PD-L1/PD-1 pathways promote the apoptosis of B cells. The secretion of GM-CSF by MSCs also inhibits the secretion of CXCR4, CXR5, IL-6, and IL-7 by activated B cells, effectively preventing the migratory ability and homing of B cells towards CXCL12 and CXCL13 chemoattractant agents and resulting in the initiation of inflammation [59]. MSCs are effective in modulating the B cell’s secreted molecules, even so, it has no negative effects on the IFN-γ, TNF-α, IL-4, and IL-10 expressions by B cells.

#### 2.2.4. Immunomodulating Effects of MSCs on NK Cells

NK cells are responsible for eliminating virus-infected cells from the host. MSCs activated by IFN-γ will upregulate the expression of HLA class I to interact with killer cell immunoglobulin-like receptors (KIRs). In this way, cytolysis mediated by NK cells is inhibited. Furthermore, toll-like receptors (TLRs) presented at MSCs are important as the activation of TLR3 increases the immunosuppression against NK cells. HLA-G will also interact with KIR2DL4 to modulate the function of NK cells [59]. Last but not least, IDO and PGE2 inhibit NK response with aid from TGF-β1 and HGF molecules.

## 3. Regenerative Mechanisms of MSCs Post-COVID-19 Infection

The unique ability of tissue regeneration and multipotent differentiation of MSCs contributes significantly to reversing the tissue damage caused by bacterial and viral infection. This ability has developed a great interest in utilizing MSCs as therapeutic agents for a wide range of chronic diseases. Apart from controlling infection and inflammation, MSCs are capable of repairing and regenerating epithelial and endothelial tissues in the alveoli of the lungs. Secretion of paracrine mediators promotes the regeneration of tissue. For post-COVID-19 patients with severe scarring of the lungs, this might be beneficial in restoring the damaged lung tissues.

Without losing sight of long-COVID patients, MSCs’ regenerative ability also foreshadows them to regenerate the lung tissues. Long-COVID patients are a subset of individuals, suffering from acute SARS-CoV-2 virus infections and developing long-term symptoms that do not unravel over months. This is also known as post-acute sequelae of COVID-19 (PASC) or post-acute COVID-19 syndrome [63].

Several theories have surfaced explaining the syndrome, but it has yet to be concretely proven. Professor Akiko Iwasaki proposed three mechanisms for long-COVID. One of the proposed mechanisms eventuating with long-COVID is the lingering virus or its reservoirs that remain within the individual’s body, leading to the ability to induce chronic inflammation [64]. Viruses can loiter somewhere else inside the host body, not necessarily in the nasopharyngeal tract. This makes delusion where the virus is thought to disappear from the lungs. As a matter of fact, they are physically present in the body, slowly and indolently driving the immune system and leading to the continuation of symptoms of COVID-19. This manifestation brings prolonged damage to the alveolar and capillary epithelial cells, which lead to edema and ARDS, not only for acute COVID-19 infection but in long-COVID patients too [65].

Long-term infection leads to lingering symptoms such as insomnia, fatigue, and difficulties in breathing. To improve the condition, the regenerative properties of the MSCs as a new and rapidly developing stem cell technology are necessary for providing an up-and-coming tissue regeneration strategy for lung cell therapy of COVID-19.

### 3.1. Regenerative Mechanism on Damaged Organ Due to COVID-19

**Figure 3:**
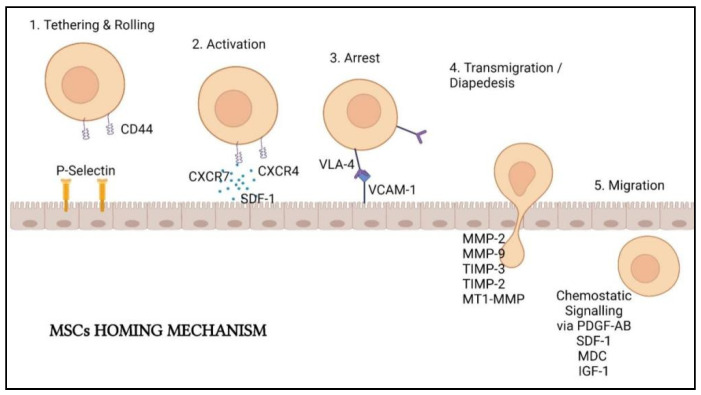
MSCs homing mechanism and involved biomolecules. Created with BioRender.com.

MSCs must first be introduced into the body to perform lung cell regeneration therapy. There are two principal methods in delivering them: local delivery (via scaffold embed or local injection) and systemic delivery (by vascular route). The delivery method is determined by the mechanism of action of the MSCs being utilized [66]. In lung cell therapy, MSCs are often intravenously (IV) injected. The injected MSCs will first entrap in the lungs a few days after injection and only migrate after a few days [67]. As inflammation and injuries happen in the lungs of COVID-19 infected patients, the entrapment of MSCs in the lungs allows homing of the stem cells to the injury sites with response to the inflammatory mediator’s gradients [68]. Several sequential steps are involved in MSCs homing: (1) tethering followed by direct rolling contacts with endothelial cells; (2) activation of integrins that are mainly induced by chemokines; (3) integrin-dependent firm adhesion to endothelial cells; (4) trans-endothelial migration and (5) interstitial migration toward the injured tissue (Figure 3) [69].

According to Yagi et al. [70], homing involves the cascade events initiated by shear resistant adhesive interactions between the flowing cells and vascular endothelium at the targeted tissues. The “homing-receptors” expressed on circulating cells interacted with appropriate endothelium coreceptors via a P-selectin-dependent mechanism, resulting in cell tethering and rolling contacts at the endothelial surface [71]. Selectins expressed by endothelial cells and CD44 expressed by MSCs are responsible for cell tethering. This interaction facilitated MSCs rolling along the wall of the organs [72].

After tethering and rolling of MSCs, the chemokine-triggered activation of integrin adhesiveness occurs. This step is facilitated by G protein-coupled chemokine receptors in response to inflammatory signals. Stem cell-derived factor (SDF)-1 on the endothelial cells will act as the ligands of the chemokine receptor CXCR4 expressed by the MSCs. Other molecules such as CXCR7 and CCR2 also facilitate MSCs homing on the endothelial cells. SDF-1 will recruit the signaling molecules to the cytoplasmic domain of VLA-4, resulting in a confirmation shift with a higher affinity for the arrest step later [73].

Integrins are important in the activation-dependent arrest of the third step of homing. Integrin 1 and 4 subunits combining will create a very late antigen 4 (VLA-4) that can interact with vascular cell adhesion molecule 1 (VCAM-1) to allow arrest followed by solid adherence [71,72]. These cellular adhesion molecules are vital for cell-based therapy because they mediate implanted cells’ adherence to the extracellular matrix of specific tissues in the host. MSCs homing was demonstrated to be abolished by inhibiting the integrin 1 subunit [74].

The penultimate phase in MSCs homing is transmigration, also known as diapedesis, and interstitial migration. MSCs must pass through the basement membrane located beneath the endothelial cells and require the cleavage of connective tissue components by different matrix metalloproteinases (MMPs). Gelatinases MMP-2 and MMP-9 are important in this stage as they are proficient in preferentially breakdown collagen and gelatin. Tissue inhibitor metalloproteinase 3 (TIMP-3), TIMP-2, and membrane type 1 MMP were also found to regulate this process (MT1-MMP). More molecular interactions are thought to be involved in MSCs extravasation, although the mechanism is yet to be comprehensible [72,75].

MSCs will then move to the injury site guided by chemotactic signals. The growth factors platelet-derived growth factor-AB (PDGF-AB), insulin-like growth factor 1 (IGF-1), and the chemokines MDC and SDF-1 all assist the movement of MSCs [73]. Inflammation cytokines such as interleukin (IL-8) also drive the MSCs migration and boost the secretion of regenerative substances such as vascular endothelial growth factor (VEGF).

Stem cell population preservation and niche restoration are necessary [76]. The establishment of stem cells in a niche enables the regulation of cell tissue generation, maintenance, and repair. Apart from preventing the stem cells from depleting, a niche also provides signals to maintain the stem cell state. MSCs can be critical in the implementation of this process. MSCs first differentiate into niche components appropriate to the tissue. Then, it attracts functional cells to the niche in the wounded areas of the lungs. The stem cell pool is then replenished by MSCs endowing differentiated cells with stemness. Because of their remarkable plasticity and ability to respond to metabolic, mechanical, and biological paracrine cues in the microenvironment, MSCs can substantially contribute to tissue regeneration. Therefore, various mechanisms are involved in the regeneration mechanism of MSCs in generating new tissues for damaged organs.

### 3.2. Mechanism of MSCs Tissue Regeneration in Lung Cell Therapy

**Figure 4:**
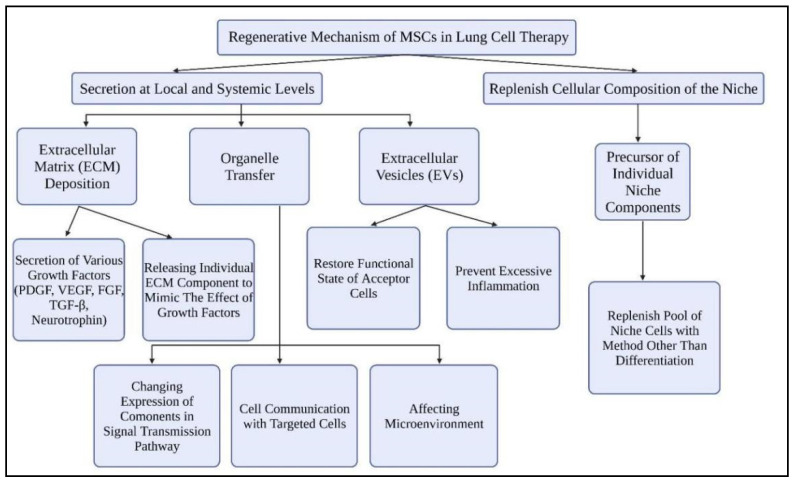
Summary of the regenerative mechanism of MSCs in lung cell therapy.

The regeneration of lung tissues by MSCs occurs via two mechanisms: secretion at local and systemic levels and restoring the cellular component into a niche (Figure 4).

#### 3.2.1. Secretion at Local and Systemic Levels

MSCs increase the secretion of various growth factors in the extracellular matrix (ECM) in response to diverse damage-related signals. When the endothelium wall of the lungs is damaged, MSCs will separate and release type I collagen. The response to signals in controlling wound healing with fibronectin will be mediated by the secreted type I collagen. Transforming growth factor beta 1 (TGF-1) is also a type I collagen response that is important for tissue healing after damage. By attaching to molecules such as platelet-derived growth factor (PDGF), vascular endothelial growth factor (VEGF), fibroblast growth factor family (FGF), TGF-β1, and neurotrophin, fibronectin will also aid in the transfer of signals to stem cells for retaining their biological activity [76].

Furthermore, MSCs also release individual ECM components that can behave as agonists or antagonists of tyrosine kinase receptors (TKR). These components act by mimicking the effects of growth factors. The secreted growth factors will be stored within the ECM and only active when tissue damage occurs. For example, epidermal growth factor receptor (EGFR) and TKR promote interstitial lung diseases. The overexpression of EGFR will stimulate pulmonary fibrosis development, which is unfavorable for COVID-19 patients [77]. MSCs secrete laminin 5 will bind with EGFR to trigger cell differentiation. Decorin, alternatively, will inhibit the signaling of these receptors. The filling of these biomolecules within the ECM will regulate the stem cell behavior in tissue repair. Eventually, they will localize to the surroundings of the MSCs and enable the reception of various paracrine signals of different nature.

MSCs also release extracellular vesicles (EVs), primarily exosomes with varying internal compositions based on external cues for lung tissue regeneration [76,78]. This is necessary for MSCs to communicate with targeted cells and convey various types of substances such as lipids, proteins, and nucleic acids. By altering the expression of components in the Wnt, PDGF, and TGF-signaling pathways, released EVs are also able to regulate the stem cell pool’s maintenance. Furthermore, the EVs secreted have an impact on the tissue’s microenvironment. MSCs released EVs containing microRNAs that suppress the production of myofibroblasts and the development of pulmonary fibrosis by reducing TGF-2, according to a study by Fang et al. [79].

MSCs mediate tissue regeneration via transferring specific organelles such as mitochondria by vesicular or tunnel nanotube transport, in addition to paracrine factors and EVs [76]. Mitochondrial transfer is triggered by damage-associated molecular patterns (DAMPs) in the form of mitochondrial DNA, mitochondrial proteins, or complete mitochondria from injured cells [80]. MSCs drive the restoration of the functional condition of acceptor cells and the protection of the niche stem cell pool from depletion by transferring their mitochondria to their microenvironment cells. This also avoids the occurrence of excessive inflammation. It was revealed in a model of acute lung damage that transferring mitochondria from MSCs to alveolar epithelial cells minimizes the detrimental effects of acute lung damage by restoring the activities of alveolar epithelial cells [80].

#### 3.2.2. Replenish the Cellular Composition of the Niche

MSCs could act as a precursor for specific specialty components by replenishing the pool of niche cells [76]. This technique could be particularly significant for terminally differentiated cells that do not grow in the niche. Plasticity is a property of some cells in an adult organism. It can take on a stem cell phenotype, fill a niche, and replace tissues that have been lost. In particular, bone marrow mesenchymal stem cells (BM-MSCs) can play a role in the transfer of its stem cell characteristics to differentiated type II alveolar epithelial cells if lung stem cell habitats are damaged [76,81]. It is worth noting that MSCs can not only drive niche cells to acquire stem cell qualities, but they can also give rise to stem and progenitor cells, ensuring the pool of supporting niche cells is replenished.

MSCs are considered useful cell therapy in regeneration and saving damaged organs without patients going through organ transplants. When inflammation occurs and leads to tissue damage, it will lead the MSCs to migrate to the inflamed sites and initiate its immunomodulatory effects. The occurrence of immunomodulation will supplementarily activate the regenerative mechanism of the MSCs. Therefore, MSCs can restore any level of damage spectacularly.

MSCs are recognized for their regeneration and immunomodulation capacity, which causes them to be attractive in cell therapy. However, controversy often surrounds the potential of MSCs as proinflammatory or anti-inflammatory cells. MSCs are plastic cells that will modify their phenotypes according to the engrafted microenvironment. The presence of stimuli in the microenvironment will promote the differentiation of MSCs into two different phenotypes [82]. For MSCs to be polarized and differentiated into two different phenotypes, this is a ligand-specific reaction. Chemokines and cytokines are required in the microenvironment to activate the toll-like receptors (TLR) [83]. There are two types of TLR, which will determine the immune phenotype of MSCs. The activation of TLR4 with LPS will endow MSCs with a proinflammatory phenotype, known as MSC1. Alternatively, an anti-inflammatory phenotype of MSC2 will be induced by activating TLR3 with Poly(I:C) [84]. Not only the types of TLR but the polarization of both MSCs phenotypes also required factors such as the engrafted microenvironment, priming duration and the MSC-T cell engagements timing for polarization licensing [85].

As an immunomodulator, MSC2 can immunomodulate by lowering down the activation of T cells within the host. It also lowers the inflammation in an acute lung injury associated with COVID-19 [86]. Increasing biomolecules by MSC2 such as IL-10, PGE2, and IDO promotes anti-inflammation in the host cell. They will slow down the occurrence of cytokine storms within the host’s body and prevent further scarring of the lungs. Compared to MSC1, these TLR4 priming cells will increase the molecules associated with cytokine storm, especially IL6 and IL8. The increase in IL6 will undoubtedly increase the inflammatory condition that happens within the host as vascular permeability increases and activates the coagulation system, which will fasten the spreading of inflammation. IL8 will also increase the disease’s progression by recruiting more neutrophils to the infected area and causing further tissue damage [87]. Increasing these biomolecules brings disadvantages to COVID-19 patients due to continuous lung scarring, promoting MSC2 as a more favorable cell phenotype for the immunomodulation of COVID-19 patients.

As MSCs can be isolated from different sources, they contain different differentiation potential [88]. They do not differentiate into the same cellular lineages. MSCs phenotypes with high potential to differentiate into lung epithelial cells are more preferred as a candidate. Concerning MSC-based therapy for long-COVID patients, MSCs derived from the bone marrow, umbilical cord and adipose tissue are more preferred as the candidates [81,89]. Some studies showed that human bone marrow MSCs (BM-MSCs) and adipose tissue MSCs (AD-MSCs) have a high potential to differentiate into alveolar epithelial cells. Umbilical cord MSCs also have fewer ethical issues than other stem cells, contributing to tissue regeneration in long-COVID patients. As MSC2 is preferred to be used in COVID-19 patients, these immunosuppressive MSCs will also contribute to the regeneration of lung tissues in long-COVID patients. While the immunomodulation of MSC2 happens within the host cells, it will also differentiate into new lung epithelial tissues, which will repair the lung scarring of long-COVID patients via various regeneration mechanisms (Figure 4). Furthermore, MSC2 will also impede the injury-driven responses caused by viruses and educate the macrophages in the lung microenvironment towards a proangiogenic phenotype [85]. The macrophages will increase the secretion of various growth factors to stimulate lung tissue regeneration and proliferation in long-COVID patients.

The switching of MSCs phenotypes certainly contributed to the immunomodulatory and lung cell regeneration of MSCs in both COVID-19 and long-COVID patients. However, the switching of phenotypes also brings its pros and cons. In a proinflammatory condition, switching MSCs into the MSC1 phenotype will enhance the inflammation of the patient’s lungs, opposite to the scientist’s aim of applying MSCs as an alternative therapeutic choice to the COVID-19 and long-COVID patients and detrimental to the patient. However, this proinflammatory situation favors the MSCs by allowing them to boost the release of growth factors such as IL-6, IL-8, and GM-CSF. These molecules will stimulate more neutrophils to the inflamed regions and promote inflammation activities. Chemokines, when stimulated, will also promote inflammation by attracting other lymphocytes to the site of inflammation. In this immune-boosting milieu, MSCs are exposed to insufficient amounts of IDO, which will promote T cell proliferation. A significant amount of proliferating T cells can induce many lymphocytes to damage healthy lung cells, exacerbating the patient’s condition. Although increasing the inflammatory state does not help enhance the patient’s lung cells, it does allow MSCs to transition from immune-enhancing to immune-suppressing properties. When MSCs are exposed to adequate proinflammatory cytokines, they adopt immune-suppressive phenotypes (MSC2), reducing inflammation and facilitating lung tissue repair. Facilitating lung tissue repair due to various secretion during the proinflammatory conditions by MSCs will benefit the patients for lung tissue regeneration.

## 4. Current Clinical Trials of MSCs in COVID-19 Treatment and Their Challenges

### 4.1. Clinical Trials of MSCs in COVID-19 Treatment

Several registered clinical trials and studies had begun within a short period. A growing number of clinical trials and experimental studies were initiated worldwide for COVID-19 under registration at ClinicalTrails.gov (https://clinicaltrials.gov/, accessed on 1 September 2021). Table 4 shows the completed studies related to the use of MSCs in COVID-19. Among 52 completed studies sorted out, only 18 are chosen as these studies are involved in the application of MSCs and COVID-19. There are also many ongoing registered clinical trials and experimental studies. Table 5 included the selected registered active trials, which are interventional studies at phases 2, 3, and 4, involving only adults from 18 years old and above. The patients with moderate and severe symptoms of COVID-19 and who must be hospitalized are included as the criteria. Clinical trials that involved children from birth to 17 years old are excluded from this review. Among 53 studies sorted out based on the requirements stated above, 18 trials were selected based on the latest study time duration and included in this review.

From the tables above, it was observed that the most used MSCs sources are the umbilical cord mesenchymal stem cells (UC-MSCs). This may be for several reasons: (1) umbilical cord contains a high concentration of MSCs, (2) UC-MSCs can be obtained during and after the birth, (3) non-invasive techniques are used, (4) Wharton-jelly is considered a by-product, (5) UC-MSCs has faster doubling times than other stem cells, especially embryonic stem cells, (6) UC-MSCs are safer as they are not tumorigenic and (7) they can be used as an allogenic treatment since the cells are immune evasive and express low levels of major histocompatibility complex (MHC) class 1 molecule and no MHC class II [16]. Moreover, only adult patients that are 18 years old and above of all gender are included in the trials. The patients involved must also be laboratory-confirmed with moderate or severe SARS-CoV-2 infections that are diagnosed by reverse-transcription polymerase chain reaction (RT-PCR) from any sources such as nasopharyngeal samples. Patients with compliance with SARS-CoV-2 infection severe pneumonia diagnosis standards are also included in these clinical trials. However, females with pregnancy, adults with systemic autoimmune diseases, malignant tumors, and patients participating in other clinical trials are omitted, which acts as the general exclusion criteria of the trials. Most of the studies and trials are evaluating the safety and efficiency of stem cells as a therapeutic action for COVID-19. The number of studies and trials are continuously increasing apart from the studies and trials listed above. From the above tables, there is only one study that published their trial results (Clinical Trials No.: NCT04491240). The trial measured the safety and efficiency of MSCs-derived exosomes in SARS-CoV-2 associated pneumonia therapy via inhalation. The trial consists of three groups of participants with ten participants in each group. Each group of participants is given different types of exosomes derived from MSCs during inhalation and one group will be the placebo group. The study results are summarized in the Table 6 below.

From the tables above, it was observed that the number of Asian trials is much higher than the European trials. These may be due to the outbreak of diseases from Asia and led to clinical trials as scientists are searching for effective treatment and cures for the diseases. The higher number of trials in Asia is also affected by the number of populations infected by the diseases. For example, China has a higher population, which can contribute to many ongoing clinical trials. The diseases outbreak was also first identified in Wuhan, China, which spread quickly within a short period, providing many patients for clinical trials. For Europe, as the disease outbreak was much later than the Asian country, this may cause the number of clinical trials to be lesser than the Asian country. The surge of COVID-19 cases due to different SARS-CoV-2 variants in Asia countries such as India, Indonesia, and Iran also contributes to the large number of patients included in the clinical trials. These infected patients allow the scientists to further identify the effectiveness of MSCs as a therapeutic application towards different types of SARS-CoV-2 variants. Not only that, factors such as the quality of research and the data, the costs of conducting clinical trials and data acquisition, the ease of study approval, well-trained investigators, adequate subject availability, low regulation and control of ongoing activities also influence the number of completed and ongoing clinical trials in the Asian in comparison to the Europe country. Issues such as the side effects of the application of MSCs as an alternative treatment for COVID-19 that contains many unknowns, the ethical issues of using stem cells as a therapeutical application also hindering the application of MSCs for COVID-19 and long-COVID patients. These issues will also affect the imbalance in clinical trials between Asian and European countries.

Furthermore, these are still the initial trials that include only small sample sizes. A small sample size allows quick addressing of research questions. With the current situation, it is difficult to work with large samples of COVID-19 patients. A sample size that is too large will expose more participants than necessary to risks. A lot of controls are also required to work with large samples of participants. Despite that, MSCs still contain the potential to be used as COVID-19 therapy at this outbreak time. Based on the information above, it was shown from the data that the exosomes exert a trend of improvement in lung lesion treatment. The patients who received exosomes exerted no adverse events and symptoms during and after inhalation. Furthermore, the peripheral capillary oxygen saturation (SpO2) also increased. Increased SpO2 shows the increase in the amount of oxygen in the blood, which allows a sufficient amount of oxygenated blood to be transported and supplied to all parts of the body. This will maintain the function of the lungs of COVID-19 patients, and effectively lower the number of patients that require ventilators and intensive care. In the trial, MSCs also showed significantly lower C-reactive protein and LDH levels. This indicates that MSCs are effective in avoiding inflammation and preventing increasing odds of severe COVID-19 outcomes.

### 4.2. Regulatory Control on MSCs Used in COVID-19

Stem cell therapy is a rapidly emerging technology as its unique abilities are the hope of scientists in treating various chronic medical conditions, even in COVID-19. Therefore, some guidelines are required as a regulatory control to ensure that the related procedures and experiments are conducted ethically. In Malaysia, there are no legal acts implemented for stem cell therapies as the Ministry of Health Malaysia believed that the present regulatory regime is sufficient for the stem cell technology and no effort was taken to introduce any legal framework. Therefore, Malaysia relies on guidelines alone to regulate stem cell technology [91].

“National Standards for Stem Cell Transplantation: Collection, Processing, Storage and Infusion of Hematopoietic Stem Cells and Therapeutic Cells” is one of the guidelines made by the Ministry of Health Malaysia in 2009 [92]. These guidelines will serve as a guide to all personnel involved in hematopoietic stem cell transplants. It will ensure the procedures are carried out safely and effectively. Moreover, the viability and quality of the hematopoietic stem cells and therapeutic cells that are preserved are also stated within the guidelines. Another Malaysian stem cell guideline is “Malaysian Guidelines for Stem Cell Research and Therapy” [93]. However, in terms of maintaining excellent ethical technological governance, this recommendation is relatively useless [91].

Malaysian stem cell guidelines have recently undergone revisions. This revised guideline will be consistent with the Guidelines for Stem Cell Research and Clinical Translation released by the International Society for Stem Cell Research (ISSCR) [94], 86 ISSCR being the world’s biggest society committed to the advancement of stem cell research, publishes standards that include requirements for the development of stem cell therapies. This global guideline published includes secular yet universal moral principles, leading to the proven therapeutic potential of stem cells. In the guideline, ISSCR defines the various stages of stem cell research, including laboratory research, preclinical, and clinical experiments with animal and human participants. Moreover, ISSCR also describes how to undertake stem cell treatments and research responsibly, with specific guidelines such as stem cell procurement, international cooperation, regulatory oversight, and social justice considerations [91]. Recently, the guideline was revised again in May 2021 to include new recommendations in addressing the recent scientific advances involving embryos, stem cell-based embryo models, chimeras, organoids, and genome editing [94].

Compared to Europe, stem cell sources such as human embryonic stem cells (hESC) are permitted in the research. Despite the differences in research legislation, they have united their ethical concerns about embryonic stem cell research [95]. The research is permissible when it follows the rules such as personal protection rights (protection of human life), political rights (e.g., no improper governmental action), and social and economic rights under European Union human rights laws and ethical standards [95]. Furthermore, in countries where hESC lines are permitted for research, they may be used if considered necessary, if no viable alternatives exist, and if the work is nonredundant. Only a few member nations, including the United Kingdom, Belgium, and Sweden, had implemented explicit regulations on using embryos for research by 2001. Slowly by 2008, roughly 16 European Union and Europe-associated countries passed legislation regulating stem cell research. Each country contains its own information on the current legal position, ethical and regulatory oversight. While several European countries are also passing legislation to allow stem cell research utilizing embryo-derived stem cells, the specific nature of regulation across Europe also remains complicated [95]. Nonetheless, a regulatory policy is still required to be formulated to govern this technology to prevent any sensitive issues in every country. This is attributed to the unregulated condition of stem cells technology, which may hinder scientists from undertaking research such as human embryos, involving ethical issues. A good example of other countries such as Thailand and Indonesia, they initiated the efforts to effectively regulate the stem cells technology within their country similar to the other developed countries, including both research and transplantation. For that reason, only with a firm and trusted policy will MSCs based therapies become safer for patients and migrate one step closer to a broader clinical utility. This will remain as the main thrust of empowering the guideline for compliance among the stakeholders.

### 4.3. Safety and Efficacy of MSCs for COVID-19 as a Therapeutic Approach

MSCs are safe and efficient as a therapeutic approach for COVID-19. There are various studies and clinical trials that were conducted to show that it was efficient in improving the lung conditions for post and long-COVID-19 patients. Leng et al. [96]. conducted a study that involved COVID-19 patients and MSCs shows that MSCs have therapeutic efficiency in patients with ALI, ARDS caused by COVID-19. In the trials, seven COVID-19 patients received ACE2-expressing bone marrow MSCs, showing a significant improvement in lung function and symptoms within two days of treatment. At 3 to 6 days of treatment, the count of peripheral blood lymphocytes has increased. C-reactive protein (CRP) levels also decrease, indicating that the level of inflammation decreased. From the patients, the immune cells such as CXCR3 + CD4+ T cells, CXCR3 + CD8+ T cells and CXCR3+ NK cells also decreased in number and disappeared. Furthermore, there are significantly lower serum TNF-α and higher serum IL-10 levels in the patients observed. From these results, it is promising that MSCs give therapeutic prospects for COVID-19 patients.

**Figure 5:**
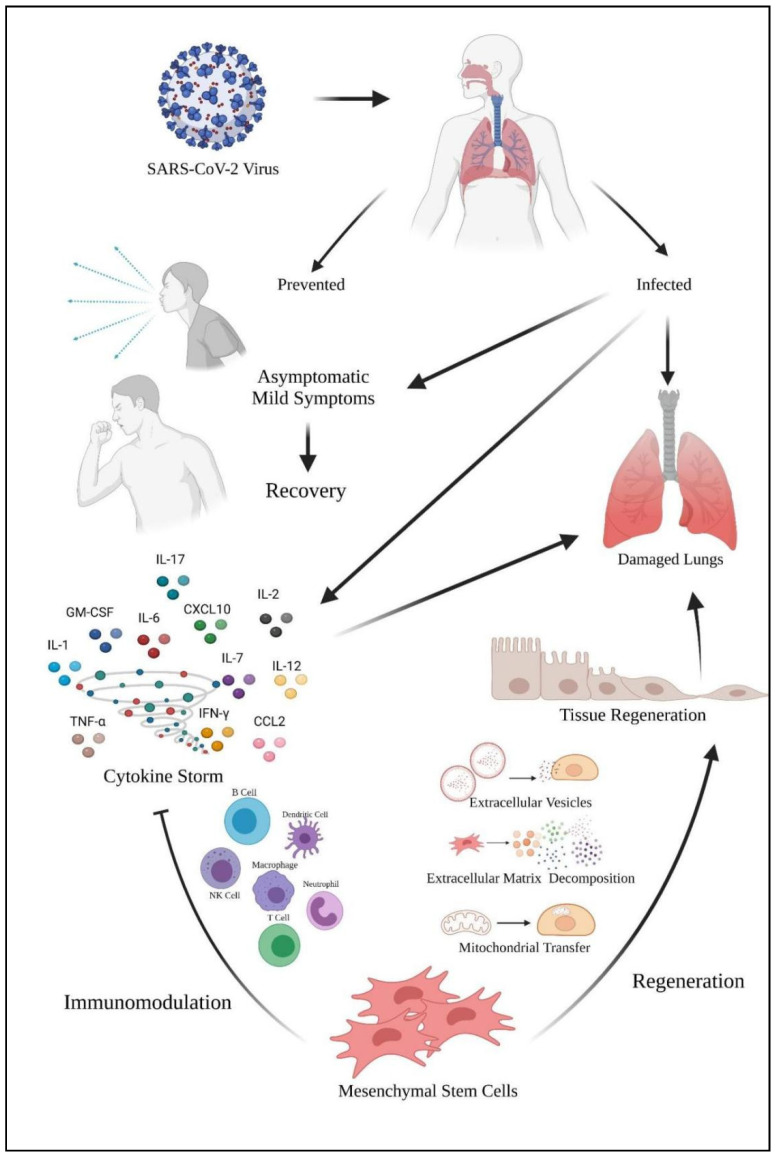
Immunomodulation and regeneration properties of mesenchymal stem cells in coping with cytokine storm caused by SARS-CoV-2 virus. Arrow represents the causes of the SARS-CoV-2 virus to the host’s lung damage and the promotion of regenerative mechanism by mesenchymal stem cells in repairing the lung. The blunt end arrow represents the inhibition of cytokine storm via various leukocytes by mesenchymal stem cells.

Figure 5 shows the role of mesenchymal stem cells in coping with the SARS-CoV-2 virus. The virus enters the host via the nasopharyngeal tract. When the infected host shows no and mild symptoms, it will lead to recovery. However, the virus will cause damage to the host’s lungs. Furthermore, the virus also caused a cytokine storm in the lungs and leads to lung damage too. Mesenchymal stem cells (MSCs) can act as a therapeutic approach in coping with cytokine storms. MSCs exhibit their immunomodulation properties via various leukocytes to calm the cytokine storm. MSCs also exhibit their regenerative mechanism to the lung tissues via various mechanisms for tissue regeneration. The tissue regeneration will contribute to lung repair which will benefit the COVID-19 patients.

## 5. Conclusions

Using stem cell-based therapies is certainly a promising alternative for this newly emerging disease, which currently has no cure. In this review, we discussed how MSCs can play a crucial role in reducing the severity of COVID-19 and possibly treat patients who suffer from long-COVID. The immunomodulation properties of MSCs provide a promising feature in inhibiting a cytokine storm and lung inflammation. As for regenerative properties, MSCs can differentiate into various cell lineages. The regenerative properties of MSCs can benefit patients in the restoration of damaged lungs scarred by the infection. This has garnered interest from researchers on cell-based therapy especially when a cure is yet to be made available.

Nonetheless, despite the positive outcomes of different MSCs research and trials, several safety concerns and downsides to this therapeutic application should be considered. MSC-treated tissues with encapsulated structures were reported to have calcification and ossifications. This structure indicates that transplanted MSCs can develop into undesired cells, which may be malignant when exposed to the local tissue milieu. Furthermore, when compared to healthy MSCs donors, MSCs generated from patients with inflammatory diseases had considerably reduced proliferation and differential potential, impacting the therapeutic qualities in COVID-19 patients. The hazards of allogenic MSC-induced immune responses must also be considered. The various MSCs expressions may evoke a solid allogeneic immunological response, exacerbating the existing tissue damage. The number of MSCs that will be able to be used in the treatment is also a concern when it comes to therapeutic application for long-COVID patients. Alternatives should be implemented to ensure that the appropriate cell number is applied for therapy that can benefit the patients. Concerns such as the ability of MSCs to successfully engraft should not be overlooked too.

As the pandemic only started late in 2019, we expect more results from research and clinical trials which would elucidate the benefits of MSCs in COVID-19 patients. By determining the criteria of the eligible patients, these trials may allow the discovery of the new treatment for COVID-19 within a short time. Furthermore, this treatment may be a low-cost alternative compared to the treatment via a combination of different types of drugs that are not cost-effective to the patients. As MSCs can be obtained from many sources, the high availability will reduce the cost of the treatment. Even though the findings towards this type of therapy have been reduced, MSCs trials should be continued. The trials may contribute to a better understanding of MSCs and allow the discovery of new stem cell-based treatment to prepare for future diseases.

### Future Perspectives

Stem cells have been studied extensively for a long time for their therapeutic ability in various diseases. It is vital to identify and comprehend therapy strategies at this critical moment so that they can be implemented successfully and cost-effectively for every sector. MSCs have a clear two-way immunoregulation ability and they can control the balance of a patient’s immune system by secreting anti-inflammatory substances. MSCs, on the other hand, can be homing into damaged tissues and secreting different growth factors to ameliorate the microenvironment, repair damaged cells, and drive cardiovascular development, which has indirect antiviral effects. As a result, the mechanism of MSCs must be thoroughly understood to avoid uncontrollable events.

MSCs can be used as a standalone immunomodulatory drug for COVID-19 patients. MSCs are well suited considering their mechanism of action is via their immunomodulating properties, significantly dampening the host’s immune response and slowing down cytokine storms within the host and lung cell destruction. MSCs as multipotent cells also contain regenerative properties, which will help to stimulate the regeneration of lung tissues. This application might be beneficial for post-COVID-19 patients with conditions such as severe lung tissue scarring and long-time symptoms. The application of MSCs as an immunomodulatory drug is similar to the concept of applying Merck antiviral drug for COVID-19, known as Molnupiravir as a treatment for adult COVID-19 patients. Molnupiravir is an investigational oral antiviral medicine released by Merck & Co., Inc., which significantly reduced the COVID-19 hospitalization and death rate by 50% [97,98]. This drug acts by inhibiting the replication of COVID-19 causative agents, the SARS-CoV-2 virus. However, this drug is at a possible high cost, thus, suggesting the use of MSCs immunomodulatory to replace the drug’s role in providing more affordable treatment for COVID-19 patients. Alternatively, the combination of both MSCs and Molnupiravir drugs could be a holy grail treatment as this combination can further reduce the responses caused by COVID-19. Patients may suffer from side effects when using only drug treatments or increasing the dosage. Instead of using only the drugs and increasing the dosage, the combination of both the MSCs and the drugs will possibly further reduce the COVID-19 response and bring the hospitalization and death rate of the COVID-19 patients down lower.

Moreover, with a combination of both 2D and 3D technologies, modification of MSCs can be carried out to enhance their proliferation and survival to treat various chronic diseases. MSCs that have been cultured for a long time will eventually senescence, which will result in the loss of their proliferative activity [99]. To deal with the problem, numerous attempts are required to raise the expression of stemness-related genes of MSCs, showing their positive qualities that can be preserved. Even if the stemness of the MSCs is not able to be enhanced, prolonging the in vitro expansion capacity is acceptable too. Cutting-edge biomedical technologies such as gene therapy or monoclonal antibody medicines also can be considered for combinatorial treatment in modifying MSCs for COVID-19 patient’s treatments.

Even though stem cell therapy is a promising therapeutic approach, it is still a long way off being broadly used clinically. As a therapeutic approach, our knowledge of MSCs’ abilities and outcomes in therapy is still relatively limited. Although there is an increase in stem cell therapy research for COVID-19, there is still limited data due to the small number of patients receiving this cell-based therapy. Therefore, further studies with larger enrollments are necessary to validate its efficacy for COVID-19.

## Figures and Tables

**Table 1:** The classification of SARS-CoV-2 variants as of 31 August 2021.

WHO Label	Pango Lineage	First Documented Date	First Documented Location
**Variants of Concern (VOC)**			
α (Alpha)	B·1·1·7	September 2020	United Kingdom
β (Beta)	B·1·351 B·1·351·2 B·1·351·3	May 2020	South Africa
γ (Gamma)	P·1 P·1·1 P·1·2 P·1·4 P·1·6 P·1·7	November 2020	Brazil
δ (Delta)	B·1·617·2 AY·1 AY·2 AY·3 AY·3·1	October 2020	India
**Variants of Interest (VOI)**			
ε (Epsilon *)	B·1·427 B·1·429	March 2020	United States of America
ζ (Zeta *)	P·2	April 2020	Brazil
η (Eta)	B·1·525	December 2020	Multiple Countries
θ (Theta *)	P·3	January 2021	Philippines
ι (Iota)	B·1·526	November 2020	United States of America
κ (Kappa)	B·1·617·1	October 2020	India
λ (Lambda)	C·37	August 2020	Peru
μ (Mu)	B·1·621	January 2021	Columbia

* SARS-CoV-2 variants of Epsilon (ε), Zeta (ζ) and Theta (θ) are currently known as former Variants of Interest (VOI).

**Table 2:** An overview of cytokines involved in cytokine storm by COVID-19.

	Sources	Actions	Results in COVID-19 Patients	Reference (s)
**Interleukins (IL)**				
IL-1	Macrophages Activated monocytes Dendritic cells B lymphocytes Neutrophils Synovial fibroblasts	Activates the secretion of other proinflammatory cytokines (IL-6 & TNF-α). Activate T helper (Th) 1 cell function. Recruit more neutrophils and monocytes to the site of infection. Induced secretion of IL-1β in monocytes and macrophages.	Destruction of lung cells and loss of pulmonary functions by increasing the viral load. Lung damage and increasing mortality risks (IL-1α). Lung cell pyroptosis, tissue damage in airway inflammation, results in fever, pain, vasodilation, and hypotension in patients (IL-1β). Secretion of pulmonary inflammation molecules resulting in extensive lung involvement (Th1 cells).	[29,34,35]
IL-2	T cells	Promotes the proliferation and activation of T, B, and NK cells. Generate effector and memory T cells.	Prevent pulmonary edema and acute lung microvascular injuries (NK cells). Prevents scarring of the lungs, leading to interstitial pneumonia, severe respiratory insufficiency of patients, and vascular leak syndrome (T cells).	[34,36]
IL-6	T and B lymphocytes Monocytes Macrophages Dendritic cells Endothelial cells	Involved in inflammation, immune response, and hematopoiesis. Stimulates the growth and differentiation of B lymphocytes and increases the generation of platelets. Stimulate T cells dysfunctionality. Activate the coagulation system and increase vascular permeability.	Formation of rheumatoid factor and other autoantibodies resulting in scarring within the lungs. T cells capacity in relation to dendritic cells is damaged. Coagulation system provides a condition for the rapid spread of inflammation.	[29,34,37,38,39]
IL-7	Epithelial cells	Activates T cells. Increase the secretion of other proinflammatory cytokines. Negatively regulates TGF-β.	T cells activated will induce anti-apoptotic BCL-2, promoting longer T cell proliferation. Prevent induced bronchial asthma (downregulation of TGF- β).	[34,40]
IL-10	Regulatory T cells Th9 cells	Inhibiting the production of proinflammatory cytokines (IFN-γ, TNF-α, IL-1β, IL-6). Prevents dendritic cell maturation by blocking IL-12. Stimulate IFN-γ production via CD8+ T cells.	Prevent further lung tissues damage by producing immunostimulatory molecules Increasing lung cell viral resistance. Ameliorates lung tissue injury by inducing collagen production and fibrocyte recruitment into the lung.	[34,36,41]
IL-12	Dendritic cells Macrophages Monocytes B cells	Develop Th1 and Th2 cells. Induced secretion of IFN-γ by T cells and NK cells via positive feedback mechanism. Acting in synergy with IL-18.	NK cells increase the binding to vascular endothelial cells. Increase in viral load in the lung’s microenvironment. Th2 regulates the immune system of the host against infection and promotes lung tissue repair.	[18,34,36]
IL-13	Th2 cells	Induce TGF-β secretion.	TGF-β inhibits naive T cells differentiation into effector cells. TGF-β inhibits Th1 cell differentiation.	[34,42]
IL-17	Th17 cells	Elevate inflammatory process and autoimmune diseases. Induced production of antimicrobial peptides.	Tissue damage of the lungs, physiological stress, and infection. Increase in viral load and disease severity, eventually causing multiorgan failure. Aggravation of lung lesions and acute respiratory distress syndrome occur when Th17 cells increase and are high in CD8 + T cells.	[34,38]
**Colony-Stimulating Factors (CSFs)**				
Granulocyte-macrophage CSF (GM-CSF)	Fibroblasts cells Endothelial cells Epithelial cells Hematopoietic cells T cells	Stimulate the proliferation and activation of macrophages, neutrophils, and dendritic cells. Increase proinflammatory cytokine production.	Activation of macrophages allows easy antigenic presentation and phagocytosis of pathogens. Increase the number of activated and life span of neutrophils to lung tissues, leading to substantial lung tissue injury and the development of ALI. GM-CSF upregulates the expression of TLR2, TLR4, and CD14 to increase cytokine production, and induce Th17-induced inflammation response.	[34,43,44,45]
**Chemokines**				
CXCL10 (IP10)	Neutrophils Endothelial cells Keratinocytes Fibroblasts Dendritic cells Hepatocytes	Induced by IFN-γ. Regulates immune system responses by activating and recruiting leukocytes (T cells, monocytes, and NK cells) Activate Th1 cell function.	Active recruitment of leukocytes causes lung tissue damage, leading to worse disease progression. IFN-γ stimulate higher viral load in the lung microenvironment, increase disease severity, and eventually to mortality. Th1 cells lead to pulmonary inflammation and extensive lung involvement in patients by initiation of lung injuries.	[29,34,38]
CCL2 (MCP-1)	Endothelial cells Epithelial cells Monocytes Microglial cells	Associated with antiviral responses in tissue. Regulates the migration and infiltration of monocytes, T cells, and NK cells. Activate Th1 cell function.	NK cells prevent pulmonary edema and acute lung microvascular injuries. Th1 cells lead to pulmonary inflammation and extensive lung involvement in patients by initiation of lung injuries.	[29,34]
**Interferon (IFN)**				
IFN-γ	T cells NK cells Monocytes Macrophages	Participates in numerous immune and adaptive immunological functions. Elevates after the activation of Th1 cells. Promotes macrophage activation and antigen presentation.	IFN-γ upregulates the viral loads in the lung microenvironment and causes tissue damage. Enhancement of major histocompatibility complex expression, activate macrophage function, stimulate chemokine production, induce apoptosis, arrest cell cycle, and enhance Fas expression.	[29,34,46]
**Tumor Necrosis Factors (TNF)**				
TNF-α	Monocytes Macrophages T cells Epithelial cells	Mediated by IL-1β and IL-6. Decrease the T cells count. Causes poor prognosis of COVID-19. Induces macrophage activation syndrome.	Elevated TNF-α leads to apoptotic death of lung epithelial and endothelial cells, resulting in tissue damage of the lungs. Macrophage activation syndrome triggers the endothelial cells, macrophages, and neutrophils to express TF within the lungs to initiate and further augment pulmonary coagulopathy and microvascular thrombosis.	[34,38]

NK cells: Natural killer cells; TGF: Tumor growth factors; BCL-2: B cells lymphoma 2; TLR: Toll-like receptor; TF: Tissue factor.

**Table 3:** Immunomodulation mechanisms of MSCs with various immune cells.

Immune Cells	Mechanism of Actions	Implicated Biomolecules	Action Pathway	Results
Macrophages and Neutrophils	Soluble Factors	PGE 2	Activation of signal transducer activators of transcription-3 (STAT 3)	M2 macrophage phenotype switch
DCs	Soluble Factors	PGE 2	Lower the expression of CD38, CD80, CD86, IL-12, and IL-6	Inhibit DCs maturation
			CCR7–CCL21 interaction	Lowering the migratory ability of DCs
		TNF-α-stimulating gene 6	Inactivation of mitogen activated protein kinase (MAPK) and nuclear factor-kappa B (NF-κB)	Suppress DCs maturation
		HLA-G	Blocking the secretion of cytokines such as TNF-α, ΙL1-α, β, IL-6, IL-7, IL-8, IL-9, GM-CSF, and IFN-γ	Prevent the differentiation of monocytes to DCs
	EVs	miR-21-5p, miR-142-3p, miR-223-3p, and miR-126-3p	Interaction with JAG1, PDCD4, IL-12p35, downregulation of IL-6 expression	Inhibition of DCs maturation
T Cells and B Cells	Soluble Factors	PGE2	CAMP production in T cells	Downregulate the IL-2 and IL-2R expression
			Negatively regulating the phosphatidylinositol hydrolysis and the diacylglycerol and inositol phosphate (IP) production	T cells inactivation
			Orchestrating regulatory T (Treg) responses	Promote Th2 immune response
		IDO	Blocks the metabolism from tryptophan to kynurenine in combination with TGF-β1 and HGF	Suppress T cells proliferation
		NO	Activating the transcription 5 phosphorylation	Inhibition of TCR-mediated T cells proliferation and inflammatory cytokine production
		Galectin 1 and 3	Preventing the clustering of TCR via crosslink interaction mechanism	Suppress the proliferation of T cells proliferation
		HLA-G with IDO and IL-10	Suppress proliferation of T cells	Indirectly blocked the secretion of cytokine (TNF-α, ΙL1-α, ΙL1-β, IL-6, IL-7, IL-8, IL-9, GM-CSF, and IFN-γ)
	Cell–Cell Interactions	Fas/Fas ligand death signaling pathway	Downstream activation of the Fas-associated death domain and caspases	T cells apoptosis
		TNF-related apoptosis-inducing ligand (TRAIL)/death receptor (DR) signaling pathway	High production of TRAIL and binds to DR on T cells	T cells apoptosis
		Programmed death ligand-1 (PD-L1)/programmed death-1 (PD-1)	Inhibition of MAPK followed by Src-homology 2 domain containing protein tyrosine phosphatases (SHP)-1 and SHP-2 phosphorylation	Reduces T cells proliferation
NK Cells	Cell-cell Interaction	HLA class I	Upregulate the expression of HLA class I molecules to interact with killer cell immunoglobulin-like receptors (KIRs)	Inhibit cytolytic activity of NK cells
			Interacting with KIR2DL4	Inhibit NK cells and cytokine production
		Toll-like receptors (TLRs)	Activation of TLR3	Increases the immunosuppression against NK cells
	Soluble Factors	IDO and PGE2 with aid from TGF-β1 and HGF molecules	Inhibit IL-2 induced NK response	Immunosuppression of NK cells

**Table 4:** Completed clinical trials of MSCs usage for the treatment of COVID-19.

Clinical Trials Identifier	Study				
	MSCs Source	Title	Outcome Measured	Trial Duration	Location
NCT04573270	UC-MSCs	Mesenchymal Stem Cells for The Treatment of COVID-19	Safety and Efficacy of Stem Cell Therapy for The Treatment of Patients Admitted to Hospital Suffering Complications from COVID-19	April 2020–September 2020	United States
NCT04288102	UC-MSCs	Treatment with Human Umbilical Cord-Derived Mesenchymal Stem Cells for Severe Coronavirus Disease 2019 (COVID-19)	Safety and Efficacy of Human Umbilical Cord-Derived MSCs (UC-MSCs) for Severe COVID-19 Patients with Lung Damage	March 2020–July 2020	China
NCT04355728	UC-MSCs	Use of UC-MSCs for COVID-19 Patients	Safety and Efficacy of Human Umbilical Cord Derived Mesenchymal Stem Cells (UC-MSCs) for Treatment of COVID-19 Patients with Severe Complications of ALI/ARDS	April 2020–October 2020	United States
NCT04492501	BM-MSCs	Investigational Treatments for COVID-19 In Tertiary Care Hospital of Pakistan	Mortality and Morbidity Benefit of Different Investigational Treatment	April 2020–July 2020	Pakistan
NCT04491240	MSCs-Derived Exosomes	Evaluation of Safety and Efficiency of Method of Exosome Inhalation in SARS-CoV-2 Associated Pneumonia.	Safety and Efficiency of Aerosol Inhalation of The Exosomes in The Treatment of Severe Patients Hospitalized with Novel Coronavirus Pneumonia (NCP)	July 2020–October 2020	Russian Federation
NCT04288102	UC-MSCs	Treatment with Human Umbilical Cord-Derived Mesenchymal Stem Cells for Severe Coronavirus Disease 2019 (COVID-19)	Safe and Effective MSCs Therapeutic Approach to COVID-19	February 2020–August 2020	China
NCT04535856	DW-MSCs	Therapeutic Study to Evaluate the Safety and Efficacy of DW-MSCs in COVID-19 Patients	Safety and Efficacy of DW-MSCs in COVID-19 Patients	September 2020–January 27 2021	Indonesia
NCT04269525	UC-MSCs	Umbilical Cord (UC)-Derived Mesenchymal Stem Cells (MSCs) Treatment for the 2019-Novel Coronavirus(nCOV) Pneumonia	Availability and Safety of UC-MSCs Treatment for Serious Pneumonia and Critical Pneumonia Caused by the 2019-nCOV Infection	February 2020–December 2020	China
NCT04252118	UC-MSCs	Mesenchymal Stem Cell Treatment for Pneumonia Patients Infected with COVID-19	Safety and Efficiency of Mesenchymal Stem Cells (MSCs) Therapy for Pneumonia Patients Infected with SARS-CoV-2	February 2020–April 2020	China
NCT04392778	UC-MSCs	Clinical Use of Stem Cells for The Treatment of COVID-19	Regenerative And Repair Abilities of Stem Cells to Fight Against the Harmful Effects of The Novel Coronavirus COVID-19	May 2020–May 2021	Turkey
NCT04898088	N/A	A Proof-Of-Concept Study for The DNA Repair Driven by the Mesenchymal Stem Cells in Critical COVID-19 Patients (Repair)	Positive Effect of Stem Cell Therapy Applied on Critically Ill Patients with Coronavirus Infection on DNA Repair Genes	January 2020–September 2020	Turkey
NCT04276987	AdMSCs-Derived Exosomes	A Pilot Clinical Study on Inhalation of Mesenchymal Stem Cells Exosomes Treating Severe Novel Coronavirus Pneumonia	Safety And Efficiency of Aerosol Inhalation of The Exosomes Derived from Allogenic Adipose Mesenchymal Stem Cells (MSCs-Exo) in the Treatment of Severe Patients Hospitalized with Novel Coronavirus Pneumonia (NCP)	February 2020–July 2020	China
ChiCTR2000029990	N/A	Clinical Trials of Mesenchymal Stem Cells for The Treatment of Pneumonitis Caused by Novel Coronavirus Pneumonia (COVID-19)	Safety And Efficacy of Human Mesenchymal Stem Cells in The Treatment of New Coronavirus (2019-nCOV) Pneumonia.	January 2020–March 2020	China
ChiCTR2000029569	UC-MSCs	Safety And Efficacy of Umbilical Cord Blood Mononuclear Cells Conditioned Medium in the Treatment of Severe and Critically Novel Coronavirus Pneumonia (COVID-19): A Randomized Controlled Trial	Effectiveness of Conventional Treatment Group and Conventional Treatment Combined with Umbilical Cord Mesenchymal Stem Cell Conditioned Medium Group in Treating Patients with Severe and Critical 2019-nCOV Coronavirus Pneumonia	February 2020–April 2020	China
ChiCTR2000031139	hESC- Derived M Cells	Safety and Effectiveness of Human Embryonic Stem Cell-Derived M Cells (CAStem) for Pulmonary Fibrosis Correlated with Novel Coronavirus Pneumonia (COVID-19)	Safety and Tolerability of CAStem Cells for COVID-19-Related Pulmonary Fibrosis	March 2020–March 2021	China
ChiCTR2000031319	DP-MSCs	Safety And Efficacy Study of Allogeneic Human Dental Pulp Mesenchymal Stem Cells to Treat Severe Novel Coronavirus Pneumonia (COVID-19) Patients	Safety and Efficacy of Allogeneic Human Dental Pulp Mesenchymal Stem Cells in The Treatment of Severe Pneumonia Caused By COVID-19, Reducing Mortality and Improving Clinical Prognosis	April 2020–July 2020	China
NCT04400032	N/A	Cellular Immuno-Therapy for COVID-19 acute respiratory distress syndrome (CIRCA-19)	Tolerability and Potential Signs of Efficacy of using MSC Therapy for Patients with Severe Infections (Sepsis) Associated with ARDS	May 2020–April 2021	Canada
NCT04382547	Mucosa-Derived MSCs	Treatment of COVID-19 Associated Pneumonia with Allogenic Pooled Olfactory Mucosa-derived Mesenchymal Stem Cells	Ability of Treatment of Severe COVID-19 Associated Interstitial Pneumonia using Allogeneic Mesenchymal Stem Cells	May 2020–June 2021	Belarus

UC-MSCs: Umbilical Cord Mesenchymal Stem Cells; BM-MSCs: Bone Marrow Mesenchymal Stem Cells; DW-MSCs: Daewoong Mesenchymal Stem Cells; AD-MSCs: Adipose Tissue-Derived Mesenchymal Stromal Cells; hESC: Human Embryonic Stem Cells; DP-MSCs: Dental Pulp Mesenchymal Stem Cells; N/A: Not Available.

**Table 5:** Active clinical trials of MSCs usage for the treatment of COVID-19.

	Study					
Clinical Trials Identifier	MSCs Source	Title	Outcome Measured	Phase	Estimated Trial Duration	Location
NCT04371393	MSCs Drug (Remestemcel-L)	MSCs in COVID-19 ARDS	Number of Days Alive Off Mechanical Ventilatory Support, Total Number of Events Over 30 Days, Number of Participants Alive and/or Improvement of ARDS, Severity of ARDS, Hospital Length of Stay, Number of Readmission and Stay in Intensive Care Unit, Change in Plasma Hs-CRP Concentration and Serum Hs-CRP Concentration, Change In IL-6, IL-8, TNF-Alpha, Inflammatory Marker Level, Pulmonary Symptoms.	3	April 2020–February 2022	United States
NCT04361942	N/A	Treatment of Severe COVID-19 Pneumonia with Allogeneic Mesenchymal Stromal Cells (COVID_MSV)	Proportion of Patients Achieved Withdrawal of Invasive Mechanical Ventilation, Rate of Mortality, Proportion of Patients Who Have Achieved Clinical Response and Radiological Responses, Blood White Cell Counts, Cellular Markers of Inflammation, Cytokines and Chemokines in Blood.	2	May 2020–December 2021	Spain
NCT04615429	N/A	Clinical Trial to Assess the Efficacy of MSCs in Patients with ARDS Due to COVID-19	Change in the PaO2/FiO2 Ratio, All-Cause Mortality, SOFA Score, Oxygen Therapy-Free Days, Duration of Hospitalization and ICU Admission, Incidence of Non-Invasive Ventilation, Survival Rate.	2	September 2020–January 2022	Spain
NCT04625738	Ex-vivo expanded WJ-MSCs	Efficacy of Infusions of MSCs From Wharton Jelly in the SARS-CoV-2 (COVID-19) Related Acute Respiratory Distress Syndrome	Efficacy of WJ-MSCs on Respiratory Function Evolution, The Duration of Invasive Mechanical Ventilation During the Hospital Stay, The Evolution of Organ Failures, The Duration of Stay in Intensive Care Unit, The Mortality During Intensive Care Unit and Hospitalization, The Evolution of Viral Load, The Immediate or Delayed Tolerance Following the WJ-MSCs Injection.	2	November 2020–August 2022	France
NCT04905836	COVI-MSCs	Study of Allogeneic Adipose-Derived Mesenchymal Stem Cells for Treatment of COVID-19 Acute Respiratory Distress	Safety and Preliminary Efficacy of COVI-MSCs, All-Cause Mortality Rate, Incidence of All Adverse Events (AEs), Number of Ventilator-Free Days, Number of ICU days, Change in Oxygenation PaO2:FiO2 Ratio.	2	October 2021–March 2022	United States
NCT05017298	Autologous AD-MSCs	Clinical Study for Subjects With COVID-19 Using Allogeneic Adipose Tissue-Derived Mesenchymal Stem Cells	Safety of AdMSCs Injection, The Mortality Rate, Immune Measurements, Organ Functional Tests, Duration Weaning from Mechanical Ventilation, ICU Monitoring, Vasoactive Agent’s Usage, Hospitalization, Mortality rate.	2	November 2021–November 2024	United States
NCT04466098	N/A	Multiple Dosing of Mesenchymal Stromal Cells in Patients with ARDS (COVID-19)	Adverse Events Related to the Infusion of MSCs, Incidence of Reduction in Biomarkers of Inflammation, Trend Changes in PaO2:FiO2 Ratio, Airway Pressure, Changes in Positive End-Expiratory Airway Pressure (PEEP), Mortality, Number of ICU-free Days, Change in Acute Lung Injury (ALI) score.	2	July 2020–December 2021	United States
NCT04869397	Allogeneic WJ-MSCs	Treatment of Respiratory Complications Associated With COVID-19 Using Umbilical Cord Mesenchymal Stromal Cells (ProTrans19+)	Rate of Use of Mechanical Ventilation, Clinical Status Evaluation Assessed, Survival, Time to Clinical Improvement, Duration of Hospitalization and ICU Stay.	2	June 2021–July 2022	Canada
NCT04428801	Autologous AD-MSCs	Autologous Adipose-derived Stem Cells (AD-MSCs) for COVID-19	Tolerability and Acute Safety of AdMSCs Infusion, IgM/IgG Antibodies Development Against SARS-CoV-2, Lymphocyte Count in White Blood Cell Counts, PaO2 Arterial Blood Gases, Mortality Rates, Change in Blood Test Values for Cytokine Panels.	2	September 2021–September 2024	United States
NCT04780685	hMSCs	A Phase II Study in Patients with Moderate to Severe ARDS Due to COVID-19	Survival, Number of Patients with Treatment-Related Adverse Events.	2	March 2021–December 2021	United States
NCT04336254	DP-MSCs	Safety and Efficacy Study of Allogeneic Human Dental Pulp Mesenchymal Stem Cells to Treat Severe COVID-19 Patients	Safety and Efficacy in The Treatment of Severe Pneumonia Caused By COVID-19, Effects in the Treatment of Severe Pneumonia of COVID-19.	2	May 2020–December 2021	China
NCT04753476	MSCs Secretome	Treatment of Severe COVID-19 Patients Using Secretome of Hypoxia-Mesenchymal Stem Cells in Indonesia	Duration of Using a Ventilator, Length of Stay from the First Treatment to Final Outcome, Recovery, Death, Routine Blood Profile, CRP, D-dimer, Blood Gas Analysis (BGA), Photo Thorax.	2	June 2020–March 2022	Indonesia
NCT04390139	WJ-MSCs	Efficacy and Safety Evaluation of Mesenchymal Stem Cells for the Treatment of Patients with Respiratory Distress Due to COVID-19	All-cause mortality, Safety of WJ-MSCs, Need for Treatment with Rescue Medication, Duration of Mechanical Ventilation, Evolution of PaO2 / FiO2 Ratio, SOFA Index, Duration of Hospitalization, Evolution of Markers of Immune Response, Feasibility of WJ-MSCs Administration, Evolution of Disease Biomarker.	2	May 2020–December 2021	Spain
NCT04445454	BM-MSCs	Mesenchymal Stromal Cell Therapy for Severe COVID-19 Infection	Safety of Intravenous Infusion of MSCs in Patients with COVID-19 Pneumonia, Efficacy of Intravenous Infusion of MSC, Effect of MSC Administration.	2	June 2020–September 2022	Belgium
NCT04390152	WJ-MSCs	Safety and Efficacy of Intravenous Wharton’s Jelly Derived Mesenchymal Stem Cells in Acute Respiratory Distress Syndrome Due to COVID-19	Mortality Difference with Treatment, Efficacy of WJ-MSCs, Safety Evaluation of WJ-MSCs, Severity of Adverse Events.	2	January 2020–April 2022	Colombia
NCT04614025	Mesenchymal-like Adherent stromal Cells (PLX-PAD)	Open-label Multicenter Study to Evaluate the Efficacy of PLX-PAD for the Treatment of COVID-19	Number of Ventilator-Free Days, All-Cause Mortality, Duration of Mechanical Ventilation.	2	October 2020–September 2022	Germany
NCT04452097	UC-MSCS	Use of hUC-MSCs Product (BX-U001) for the Treatment of COVID-19 With ARDS	Incidence of Infusion-Related Adverse Events, All-Cause Mortality, Duration of ICU Stay, Duration of Hospital Stay, Changes in Blood Cytokine Levels.	2	July 2021–March 2022	United States
NCT04602442	MSCs Exosomes	Safety and Efficiency of Method of Exosome Inhalation in COVID-19 Associated Pneumonia	Safety Assessment, Time to Clinical Recovery (TTCR), SpO2 Concentration Changes, Blood Biochemistry (CRP).	2	October 2020–December 2021	Russian Federation

UC-MSCs: Umbilical Cord Mesenchymal Stem Cells; BM-MSCs: Bone Marrow Mesenchymal Stem Cells; AD-MSCs: Adipose Tissue-Derived Mesenchymal Stromal Cells; hESCs: Human Embryonic Stem Cells; DP-MSCs: Dental Pulp Mesenchymal Stem Cells; N/A: Not Available.

**Table 6:** Summary of the results of clinical trial NCT04491240 based on their outcome measured.

Clinical Trial	Outcome Measured and Description	Time Frame	Results
NCT04491240: Evaluation of Safety and Efficiency of Method of Exosome Inhalation in SARS-CoV-2 Associated Pneumonia. (COVID-19EXO) [90]	Number of Participants with Non-serious and Serious Adverse Events During Trial. (Safety assessments such as adverse events will be registered and will be monitored during all trials)	30 days after clinic discharge	The number of participants with non-serious and serious adverse events for three groups of participants during the trial is 0.00%.
	Number of Participants with Non-serious and Serious Adverse During Inhalation Procedure. (Safety assessments such as adverse events during the inhalation procedures will be registered)	After each inhalation for 10 days	The number of participants that show symptoms * during the inhalation procedure for three groups of participants is 0.00%.
	Time to Clinical Recovery (TTCR). (Measure and compare time to clinical recovery compared to placebo)	From first inhalation until discharge from the clinic, up to 30 days	The mean of TTCR is measured in days. Groups given with EXO1 require 13.8 days (SD: 1.55) while EXO2 groups require 14.8 days (SD: 2.35). As compared to the placebo group, it only requires 14.1 days (SD: 1.37).
	SpO2 Concentration. (Peripheral capillary oxygen saturation before and after each inhalation with a total 4 measures per day)	10 days during inhalation	Group EXO-1 shows an increase in the median of SpO2 concentration from day 1 to 10 from 93.8 to 97.8. Group EXO-2 also shows an increase in the median of SpO2 concentration from day 1 to 10 from 94.5 to 98.5. For the placebo group, the median of SpO2 Concentration is 94 and increases to 98.8 on the 10th day.
	C-reactive Protein. (Blood biochemistry C reactive protein level in serum)	At the beginning of inhalation (day 1) and on the next day of last inhalation (day 11)	Group EXO-1 shows a major decrease in the mean of C-reactive Protein level from 78.3 (Day 1) to 5.04 (Day 11). Group EXO-2 also shows decreasing mean from 75.4 (Day 1) to 5.83 (Day11). For the placebo group, the mean protein level drops less than both exosome inhalation groups, which is from 61.5 (Day 1) to 8.29 (Day 11).
	Lactic Acid Dehydrogenase (LDH). (Lactic Acid Dehydrogenase (LDH) level in serum)	At the beginning of inhalation (day 1) and on the next day of last inhalation (day 11)	Group EXO-1 shows a major decrease in the mean of LDH from 773 U/L (Day 1) to 441 U/L (Day 11). Group EXO-2 also shows decreasing mean from 732 U/L (Day 1) to 365 U/L (Day11). For the placebo group, the mean of LDH drops less than both exosome inhalation groups, which is from 669 U/L (Day 1) to 430 U/L (Day 11).

* Symptoms measured: bronchospasm, allergic reaction in the form of swelling, Quincke, rash, seizures, and other symptoms. SD: Standard Deviation.

## Data Availability

Not applicable.
